# Regulation of Nasal Airway Homeostasis and Inflammation in Mice by SHP-1 and Th2/Th1 Signaling Pathways

**DOI:** 10.1371/journal.pone.0103685

**Published:** 2014-08-04

**Authors:** Seok Hyun Cho, Sun Young Oh, Andrew P. Lane, Joan Lee, Min-Hee Oh, Seakwoo Lee, Tao Zheng, Zhou Zhu

**Affiliations:** 1 Department of Otolaryngology-Head and Neck Surgery, Johns Hopkins University School of Medicine, Baltimore, Maryland, United States of America; 2 Division of Allergy and Clinical Immunology, Johns Hopkins University School of Medicine, Baltimore, Maryland, United States of America; 3 Department of Medicine, Johns Hopkins University School of Medicine, Baltimore, Maryland, United States of America; 4 Department of Otorhinolaryngology-Head and Neck Surgery, College of Medicine, Hanyang University, Seoul, Korea; Beijing Institiute of Otolaryngology, China

## Abstract

Allergic rhinitis is a chronic inflammatory disease orchestrated by Th2 lymphocytes. Src homology 2 domain-containing protein tyrosine phosphatase (SHP)-1 is known to be a negative regulator in the IL-4α/STAT-6 signaling pathway of the lung. However, the role of SHP-1 enzyme and its functional relationship with Th2 and Th1 cytokines are not known in the nasal airway. In this study, we aimed to study the nasal inflammation as a result of SHP-1 deficiency in viable motheaten (*mev*) mice and to investigate the molecular mechanisms involved. Cytology, histology, and expression of cytokines and chemokines were analyzed to define the nature of the nasal inflammation. Targeted gene depletion of Th1 (IFN-γ) and Th2 (IL-4 and IL-13) cytokines was used to identify the critical pathways involved. Matrix metalloproteinases (MMPs) were studied to demonstrate the clearance mechanism of recruited inflammatory cells into the nasal airway. We showed here that *mev* mice had a spontaneous allergic rhinitis-like inflammation with eosinophilia, mucus metaplasia, up-regulation of Th2 cytokines (IL-4 and IL-13), chemokines (eotaxin), and MMPs. All of these inflammatory mediators were clearly counter-regulated by Th2 and Th1 cytokines. Deletion of IFN-γ gene induced a strong Th2-skewed inflammation with transepithelial migration of the inflammatory cells. These findings suggest that SHP-1 enzyme and Th2/Th1 paradigm may play a critical role in the maintenance of nasal immune homeostasis and in the regulation of allergic rhinitis.

## Introduction

Constantly encountering exogenous antigens and pathogens from the environment, upper and lower airways form an effective defense while maintaining tolerance to self-antigens [1]. This mucosal immune homeostasis can become dysregulated, resulting in skewed immune responses, such as T cell mediated Th1, Th2, or Th17 responses. Allergic rhinitis and chronic rhinosinusitis with polyposis are examples of persistent inflammatory diseases of the upper airway dominated by CD4^+^ Th2 effector cells secreting IL-4, IL-5, and IL-13 in response to commonly inhaled antigens [2]–[4]. Recently, it has been recognized that Th2-dominated upper airway inflammation may lead to long-term airway remodeling [5]. In the Th1/Th2 paradigm, the Th1 cytokine IFN-γ is considered counter-regulatory to Th2 responses [6]. Various levels of IFN-γ were found in sinus lavage samples [7] and few studies have examined the direct effects of IFN-γ on eosinophilic inflammation in allergic rhinitis and chronic rhinosinusitis [8].

Src homology 2 domain-containing protein tyrosine phosphatase 1 (SHP-1) is a negative regulator of the Th2 related IL-4Rα signaling pathway. Once recruited, phosphorylated, and activated, SHP-1 binds to and dephosphorylates its target molecules and terminates the activation signaling [9]. When SHP-1 enzyme activity is absent or reduced, cytokine/growth factor signaling goes unchecked, which may lead to abnormal responses. The motheaten and related motheaten viable (*mev*) mice are natural mutant strains deficient in SHP-1 [10]–[12]. These mice develop spontaneous inflammatory disorders in multiple organs, including manifestations in the lung [10], [11], [13]–[16]. We have previously reported that *mev* mice develop a spontaneous asthma-like phenotype in the lung [17], are more sensitive to allergen sensitization and challenge [18], and develop eosinophil-prominent inflammation in the nose similar to allergen induced allergic rhinitis [19]. However, the molecular mechanisms underlying this rhinitis in *mev* mice and the roles of Th1 and Th2 cytokines in SHP-1 regulated signaling pathways have not been studied. The *mev* mice provide an excellent genetic model to study the function of SHP-1 in the development of nasal airway inflammation.

Eosinophilia in the nasal lavage (NAL) fluid and tissues is a hallmark of allergic rhinitis and chronic rhinosinusitis with nasal polyps [20]. The trafficking of eosinophils involves many components including Th2 cytokines (IL-4, IL-5, and IL-13), chemokines (eotaxin, MCPs, and RANTES), adhesion molecules (ICAM-1 and VCAM-1) and matrix metalloproteinases (MMPs) [21]. MMPs are a subfamily of zinc- and calcium-dependent enzymes and are responsible for many physiological and pathological processes [22]. Previous studies have shown increased expression of MMPs in the patients with asthma and allergic rhinitis [5], [23]. MMP-9 is highly increased in bronchial biopsies from asthmatics compared with normal subjects [24]. Tissue inhibitors of metalloproteinases (TIMPs) are the major endogenous regulators of MMP activities in the tissue microenvironment. Disruption of the fine balance between MMPs and TIMPs has been known to be involved in many diseases including asthma, arthritis and cancer [25].

In this study, we described Th2-skewed upper airway inflammation occurring spontaneously in mice deficient in either SHP-1 or IFN-γ. These experimental results suggest that in the absence of counter-regulation, there is a default Th2 cytokine predominance in the mouse nasal mucosa. This may have important implications for understanding mechanisms deriving upper airway eosinophilic inflammatory diseases, as well as factors underlying the imbalance of MMPs and TIMPs in airway remodeling associated with these conditions.

## Materials and Methods

### Animals

The motheaten viable (*mev*) mice were used in this study because these mice have milder phenotype and longer life expectancy compared to the original motheaten mice [11]. The *mev* (*Ptpn6^me-v^*) mice and IL-4 and IFN-γ-null mice on C57BL/6 genetic background were purchased from Jackson Laboratory (Bar Harbor, Maine). Heterozygous *mev* mice were bred to generate WT, heterozygous, and homozygous *mev* mice. IL-13-null mice were generated as described by McKenzie et al. as previously reported [26] and backcrossed to C57BL/6 genetic background for more than 10 generations. Crossbreeding between *mev* mice and IL-4, IL-13, or IFN-γ-null mice was performed to generate *mev* mice on respective gene KO background. Mice were used for experiments at 7 to 9 weeks of age. All mice were housed in cages with microfilters in the specific pathogen-free environment and had free access to food and water. All animal experiments were approved by the IACUC of the Johns Hopkins University.

### Nasal lavage and cytology

Nasal airway lavage (NAL) on mice and analysis of infiltrating inflammatory cells were performed using the trans-pharyngeal nasal lavaging technique developed by our laboratory [19]. Different from previously used trans-tracheal technique [27], this technique minimizes cells loss and gives consistent cytology results [19]. Briefly, the choana was cannulated with 24G catheter through the pharyngeal opening above the vocal cord by transpharyngeal approach. The nasal cavities were gently lavaged with 350 µL of cold PBS twice and the fluid from the nostrils was collected. NAL fluids were centrifuged, and supernatants were stored at −80°C until assayed. Cell pellet was resuspended with 100 µL PBS and the total cells were counted using hemocytometer. Cytospin slides were prepared and stained with Diff-Quick stain (Dade Behring, Deerfield, Illinois), followed by differential cell count of at least 200 cells per slide.

### Histological evaluation of nasal tissues

H&E and Alcian blue stains were performed on 4 µm thick, paraffin-embedded nasal tissue sections after overnight decalcification with TBD-2 and fixation with formalin solution 10% (Thermo Fisher Scientific, Ashville, NC). For histological comparison, we used the same section through the vomeronasal organ. Nasal turbinates (maxilloturbinates) were selected for assessment of inflammation and septal mucosa was the focus for goblet cell hyperplasia.

### Real-time RT-PCR analysis of gene expression in nasal mucosa

After nasal lavage, the head of the mouse was split in a sagittal plane along the suture lines in the skull and nasal septum. Septal and turbinate mucosa were collected and placed in 1 ml of RNAlater solution (Applied Biosystems, Foster City, CA) and stored in 4°C until RNA extraction. After isolation with the RNeasy Mini Kit (Qiagen, Valencia, CA), total RNA was analyzed by Real-time PCR using the Applied Biosystems StepOnePlus machine with standard cycling parameters. Taqman gene expression assay from Applied Biosystems was used to measure the expression of TNF-α, Th1 cytokine (IFN-γ), Th2 cytokines (IL-4, IL-5, and IL-13), chemokines (CCL2, CCL5), and growth factor (GM-CSF). All probes for target genes were purchased from Applied Biosystems, GAPDH (Mm99999915_g1), IL-4 (Mm00445258_g1), IL-5 (Mm01290072_g1), IL-13 (Mm00434204_m1), IL-10 (Mm00439616_m1), IFN-γ (Mm01168134_m1), CCL2 (Mm00441242_m1), CCL5 (Mm01302427_m1), and GM-CSF (Mm01290062_m1). Each PCR run was accompanied by housekeeping gene GAPDH as an internal control [28]. Amplicon expression in each sample was normalized to its GAPDH mRNA content and the level of expression of target mRNA was determined as the delta Ct (ΔCt), the difference in threshold cycles for target gene vs. housekeeping gene. Relative gene expression was expressed as the 2^−ΔCt^ as described previously [29].

### Cytokines and chemokines in nasal lavage fluids

ELISAs were used to measure concentrations of eotaxin, CXCL1 (R&D Systems, Minneapolis, MN), and TNF-α (eBioscience, Inc., San Diego, CA) in the NAL fluids from mice. The detection limit for eotaxin, CXCL1, and TNF-α was 3.0, 2.0, and 8.0 pg/ml, respectively.

### MMP-2 and MMP-9 enzyme activity in nasal lavage fluids

Gelatin zymography was performed for MMP-2 and MMP-9 detection. Gelatin (1 mg/ml) was co-polymerized into a 10% polyacrylamide resolving gel. NAL fluids (3 µg total protein per lane) from mice were then subjected to separation by gelatin-SDS-PAGE gel. Following electrophoresis, the gel was washed with buffer containing 0.1% Triton X-100, 10 mM Tris/HCl, 10 mM NaCl, 10 mM CaCl_2_, 100 µM ZnSO_4_, pH 7.4 at room temperature for 15 min three times and then incubated with the same buffer for 12 hrs to allow enzymatic gelatin substrate hydrolysis. The gel was stained in 0.1% Coomassie blue in a mixture of methanol:acetic acid:water (volume ratio of 5∶1∶4) for 1 hr and destained with a mixture of ethanol:acetic acid:water (1∶1∶8) for 12 hrs. Densitometric analyses of triplicate data were performed using the NIS-Elements AR 3.0 software (Nikon Instrument Inc., Melville, NY).

### Statistical analysis

The total and differential cell counts data were collected from multiple groups that showed normal distribution. Data were analyzed and compared with two-way ANOVA. All values are shown as Mean±Standard deviation of the mean. The mRNA expression (ΔCt), protein concentration (pg/ml) of cytokines and chemokines, and densitometric results (arbitrary units) of MMPs were compared using one-way ANOVA. Difference with *P*<0.05 was considered statistically significant. Results were analyzed with statistical software Prism 4 (GraphPad Software Inc., La Jolla, CA).

## Results

### Spontaneous eosinophilic rhinitis in *mev* mice

**Figure 1:**
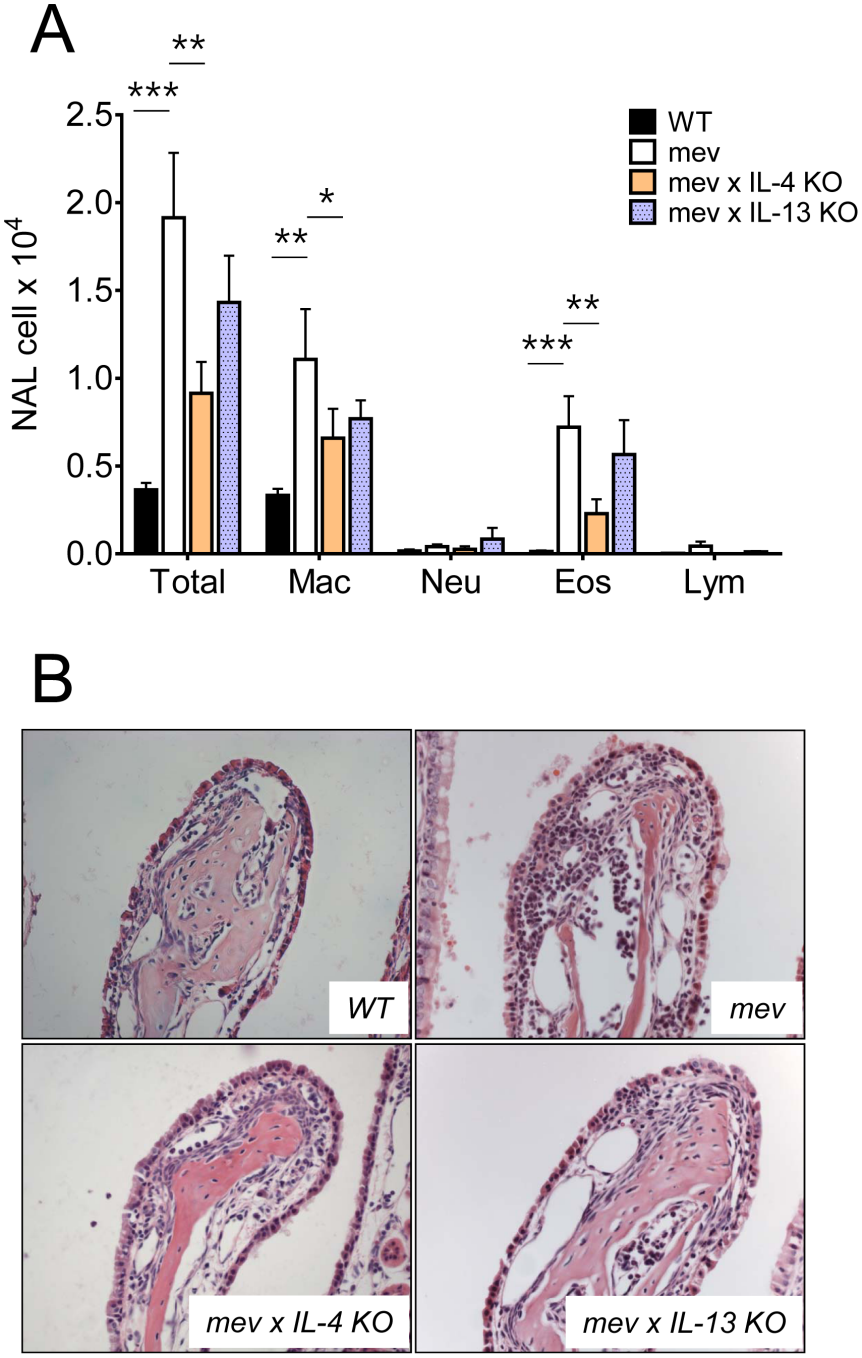
Effect of SHP-1 deficiency and role of Th2 cytokines (IL-4 and IL-13) in the nasal airway inflammation. Cytology of nasal lavage (NAL) fluid and histology of nasal airway of *WT* (n = 10), *mev* (n = 7), *mev x IL-4 KO* (n = 7), and *mev x IL-13 KO* (n = 10) mice. (A) Total and differential cell counts were done with NAL samples from each group of mice (*P < 0.05, **P<0.01, and ***P<0.001). (B) Representative hematoxylin and eosin-stained (H&E) nasal sections from each group.

First, we compared the cytology of the nasal airway lavage (NAL) fluids and nasal histopathology of WT and *mev* mice (**Figure 1A, 1B**). WT mice had normal cellularity in the nasal airway with mostly macrophages and showed no inflammatory infiltrates in the H&E stained sections. In contrast, increased cellularity with eosinophilia was readily seen in the nasal airways of *mev* mice, which is characteristic of type 2 inflammatory responses. To determine whether specific molecules in the Th2 signaling pathway plays a critical role in the nasal inflammation of *mev* mice, we selectively deleted IL-4 and IL-13 genes by crossbreeding *mev* mice with respective gene knockout (KO) strains. The results showed that *mev* mice with IL-4 gene KO had significantly decreased inflammation in the airway and in the nasal tissue (**Figure 1A, 1B**).

### Role of Th1 cytokines in the nasal airway inflammation of *mev* mice

**Figure 2:**
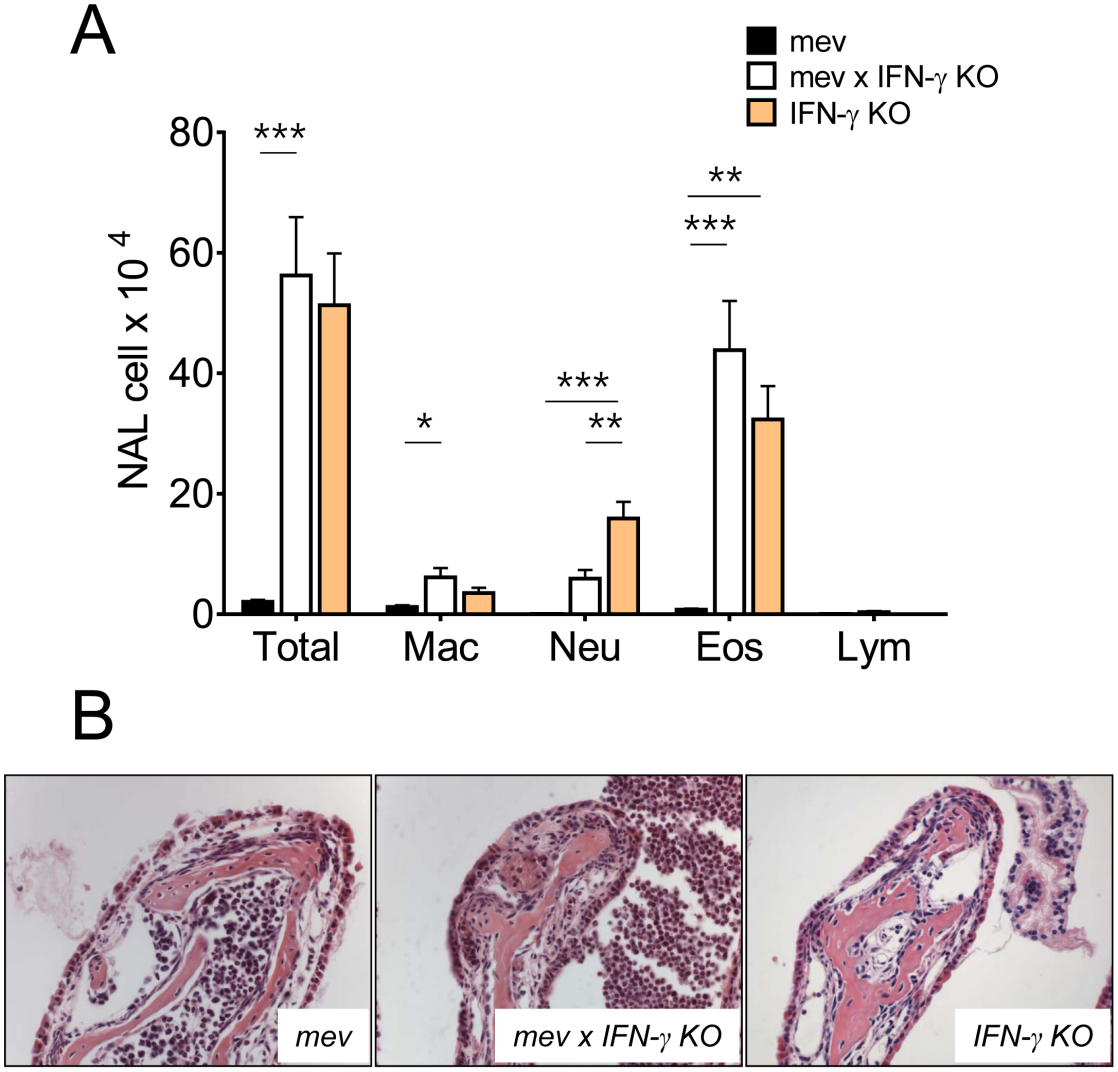
Targeted gene depletion of Th1 cytokine IFN-γ resulted in a severely Th2-skewed inflammation in the nasal airway of the *mev* mice. Histology and the nasal lavage (NAL) cytology of *mev* (n = 7), *mev with IFN-γ KO* (n = 9), and *IFN-γ KO* (n = 12) mice. (A) Total and differential cell counts in NAL samples were determined for each group of mice (*P<0.05, **P<0.01, and ***P<0.001). (B) Representative H&E-stained nasal sections from each group.

To determine the role of Th1 signaling pathway in nasal airway inflammation, we selectively deleted the IFN-γ gene by crossbreeding *mev* mice with IFN-γ KO mice. As shown in **Figure 2A**, nasal cytology of the IFN-γ KO mice showed a robust nasal inflammation with increased numbers of total cells, neutrophils, and eosinophils. However, *mev* mice on IFN-γ KO background showed significant increases in macrophages, neutrophils, and eosinophils, but not lymphocytes. Interestingly, changes in eosinophils were the most striking. As shown in H&E stained nasal sections, although some eosinophils were still infiltrated in the nasal tissues, most of the locally recruited cells moved into the nasal airway (**Figure 2B**).

### A Th2-skewed cytokine profile in the nasal cavity of *mev* mice

**Figure 3:**
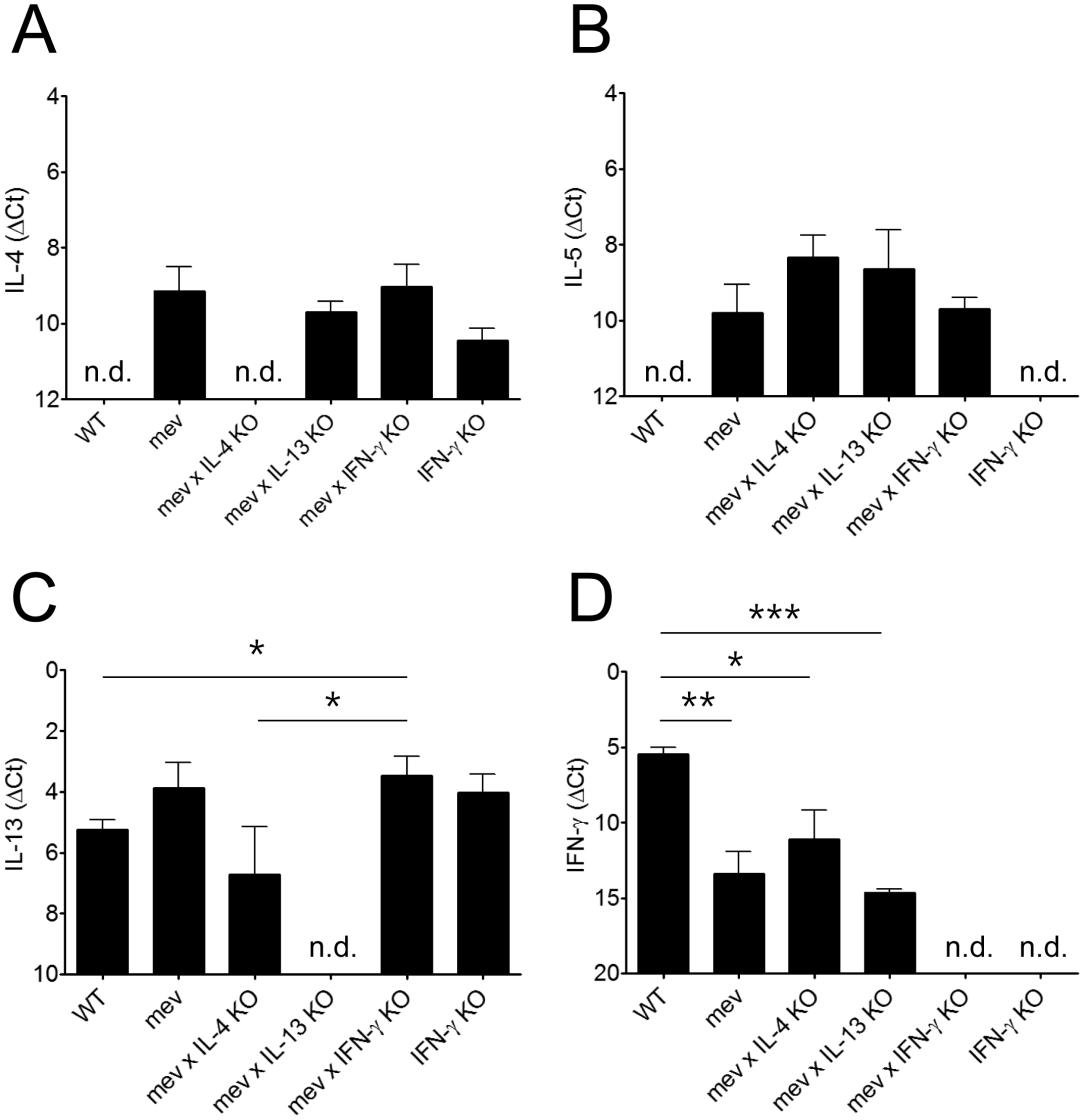
Th2 and Th1 cytokine profiles in the nasal airways of the *mev* mice. Real-time PCR showed mRNA expression of (A) IL-4, (B) IL-5, (C) IL-13, and (D) IFN-γ from *WT*, *mev*, *mev x IL-4 KO*, *mev x IL-13 KO*, *mev x IFN-γ KO*, and *IFN-γ KO* mice (n = 6 each). The results were normalized to GAPDH (as reference gene [28]) and presented as the difference of Ct values (ΔCt) between them (n.d.: not detected; *P<0.05, **P<0.01, and ***P<0.001).

We then determined the mRNA expression of Th2 cytokines (IL-4, IL-5, and IL-13) and Th1 cytokine (IFN-γ) in the nasal tissues. The mRNA levels of IL-4 and IL-5 were not detected in the nasal tissues of WT mice but were readily detected in those of *mev* mice (**Figure 3A, 3B**). In contrast to IL-4 and IL-5, there was basal level expression of IL-13 mRNA in the nasal tissues of WT mice. Increased IL-13 mRNA was seen in *mev* mice above baseline but the difference was not significant (**Figure 3C**). These findings are the opposite of the changes in the lower airways of *mev* mice where IL-13 is significantly increased [17]. Deletion of IFN-γ gene slightly but significantly increased IL-13 expression in the *mev* mice, but not in WT mice. On the other hand, IFN-γ mRNA was detected at the baseline (WT), significantly decreased in the *mev* mice, but not affected by Th2 cytokine deletions (**Figure 3D**). Thus, in the nasal airway of *mev* mice, IFN-γ deficiency slightly but significantly increased IL-13 expression but Th2 cytokine deficiencies (IL-4 and IL-13) had no effect on the expression of IFN-γ.

### Regulation of inflammatory cytokines and chemokines by Th2/Th1 cytokines

**Figure 4:**
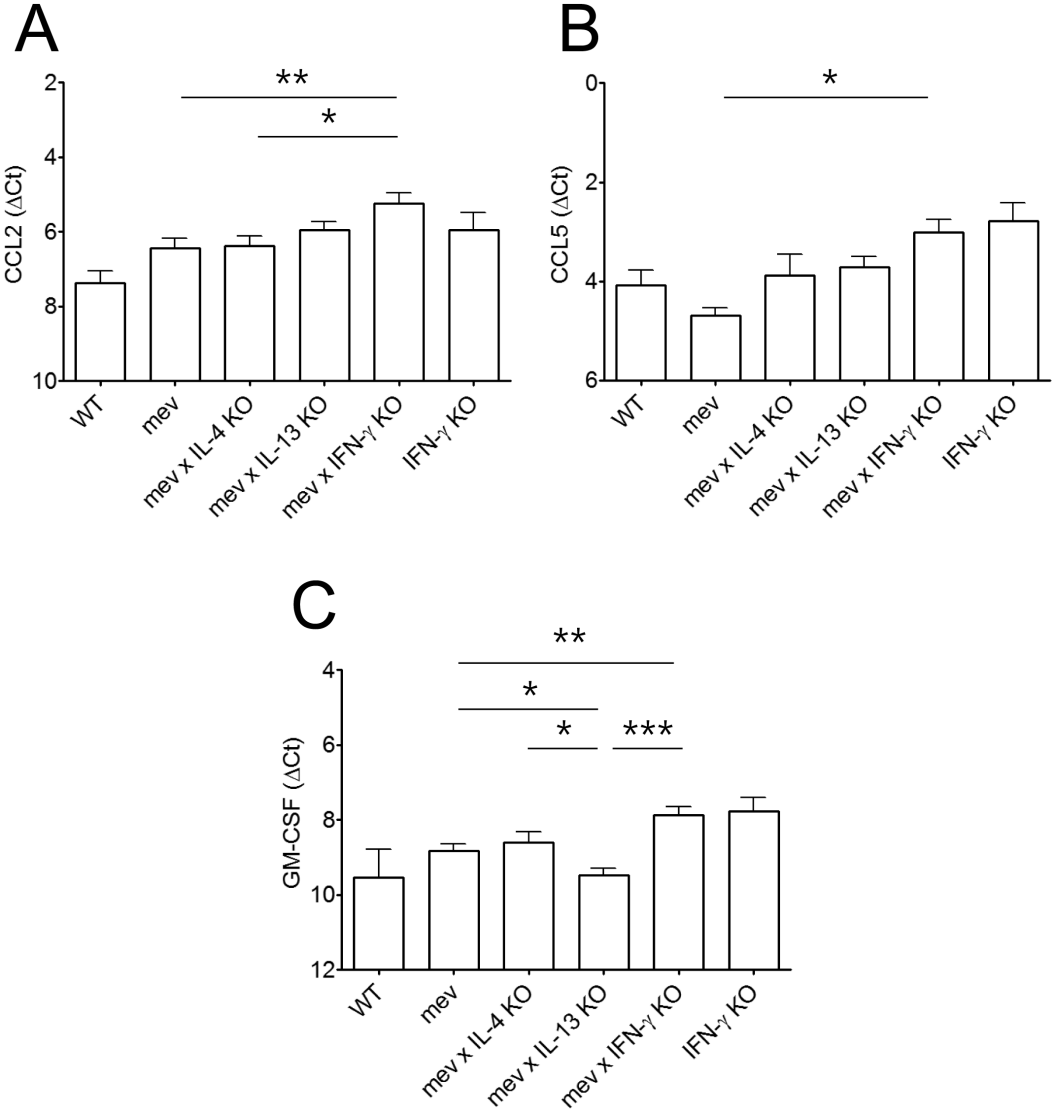
Chemokine expression in the nasal tissues of the *mev* mice. Real-time PCR showed mRNA expression of (A) CCL2 (MCP-1), (B) CCL5 (RANTES), and (C) GM-CSF from *WT*, *mev*, *mev x IL-4 KO*, *mev x IL-13 KO*, *mev x IFN-γ KO*, and *IFN-γ KO* mice (n = 6 each). The results were normalized to GAPDH (reference gene) and presented as the difference of Ct values (ΔCt) between them (*P<0.05, **P<0.01, and ***P<0.001).

To further understand the nasal inflammatory responses at the molecular level, we examined the chemokine expression in the nasal tissues of WT and *mev* mice using RT-PCR and ELISA. The mRNA levels of CCL2 (MCP-1), CCL5 (RANTES), and GM-CSF did not increase in *mev* mice as compared with those in WT mice (**Figure 4**). Expression of GM-CSF decreased in *mev* mice on IL-13 KO background. CCL2, CCL5 and GM-CSF all significantly increased in the *mev* mice with IFN-γ KO when compared with *mev* mice. However, deficiency of IL-4 did not affect the expression levels of these chemokines. Similar results were seen for CCL2 and CCL5 in IL-13 deficient *mev* mice but GM-CSF was significantly reduced in the absence of IL-13 (**Figure 4C**).

**Figure 5:**
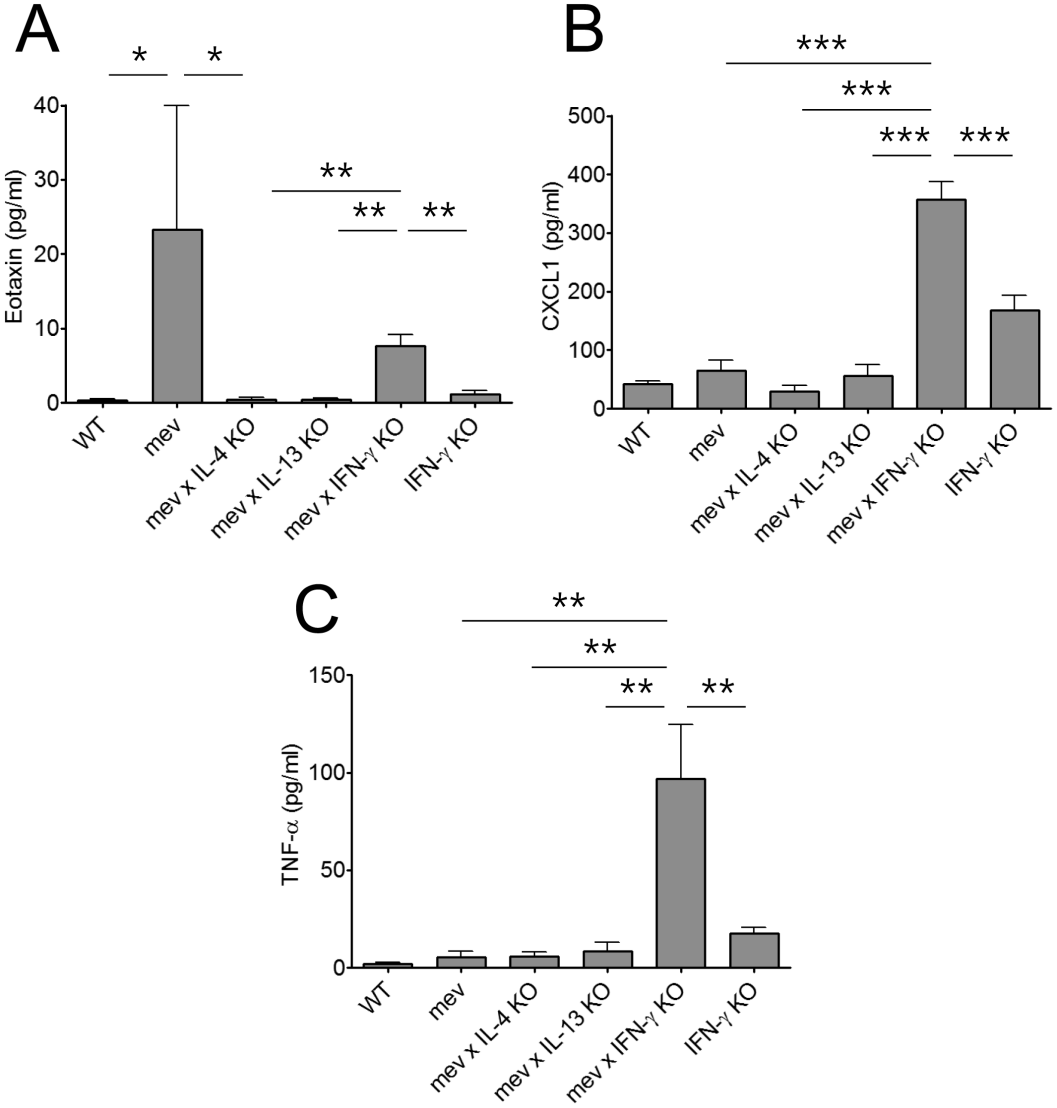
Chemokine production in the nasal lavage fluids of *mev* mice. ELISA showed proteins concentrations of (A) Eotaxin, (B) CXCL1, and (C) TNF-α from *WT*, *mev*, *mev x IL-4 KO*, *mev x IL-13 KO*, *mev x IFN-γ KO*, and *IFN-γ KO* mice (n = 6 each, and *P<0.05, **P<0.01, and ***P<0.001).

We next measured the protein concentrations of eotaxin, CXCL1 and TNF-α using ELISA. The eotaxin concentration in the NAL fluids was significantly increased in the *mev* mice compared to WT mice and this change was totally dependent on Th2 cytokines IL-4 and IL-13, and to a large extent, on IFN-γ (**Figure 5A**). In contrast, the levels of CXCL1 and TNF-α did not change in *mev* mice compared to WT mice. However, deletion of the IFN-γ gene dramatically increased the expression of CXCL1 and TNF-α in *mev* mice (**Figure 5B, 5C**).

### Increased expression and enzyme activity of MMP-2 and MMP-9 in nasal inflammation

**Figure 6:**
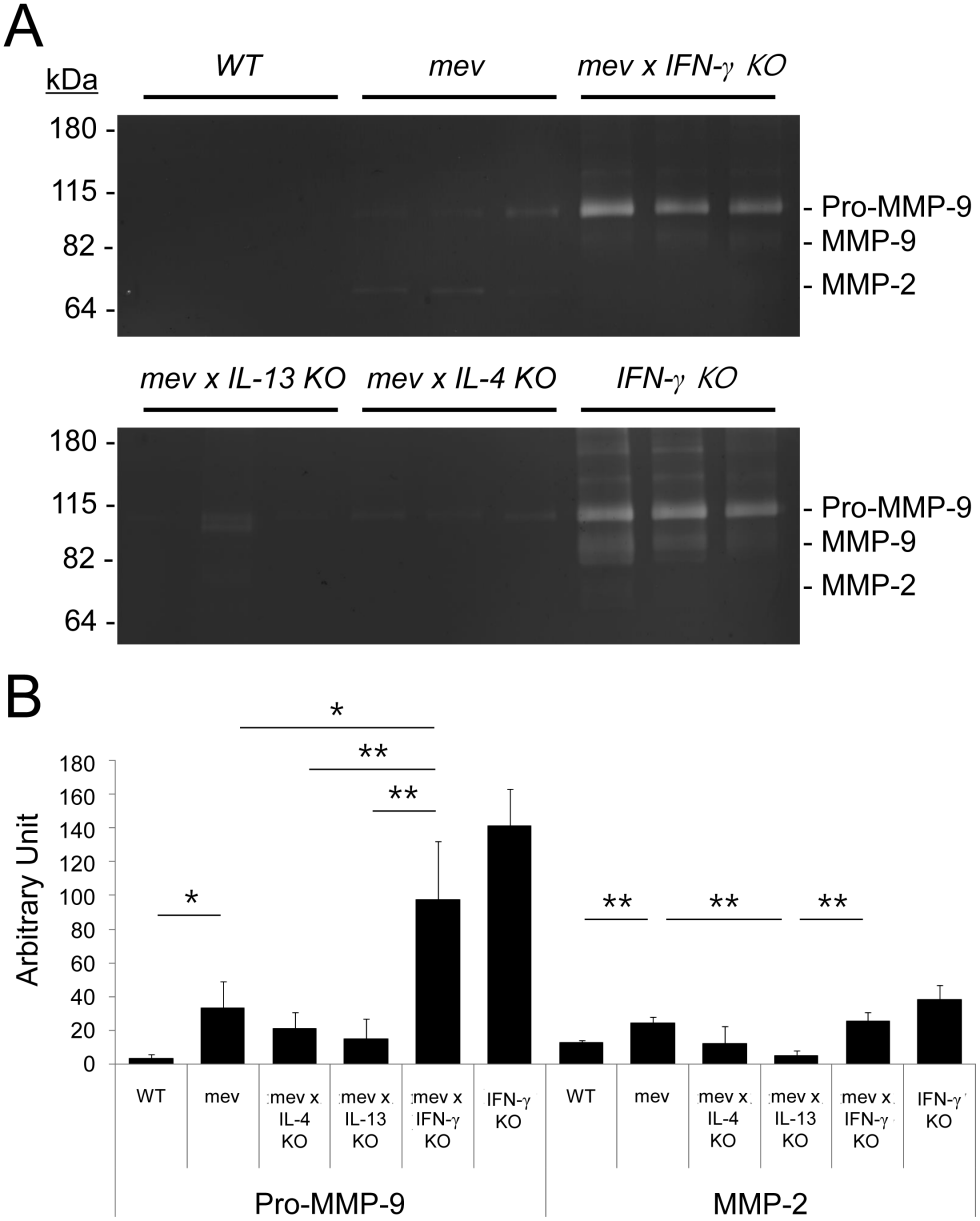
Gelatin zymography for MMP-2 and MMP-9 activities in the NAL fluids of *mev* mice. (A) MMP activity was visualized as clear bands against a dark blue background of gelatin substrate in gel. Molecular weight markers were used to estimate the molecular masses of the pro and active forms of MMP-2 and MMP-9. Expression of MMP-9 was identified as mainly pro-forms instead of minor active forms. Expression of MMP-2 was seen as active form without pro-form. MMP-9 expression was more prominent than MMP-2 in the NAL fluids. (B) Blots were scanned and densitometry was performed. Density of each band expressed as an arbitrary unit. This gel is a representative of two independent experiments.

We measured the expression and enzyme activity of MMP-2 and MMP-9 in the NAL fluids by gelatin zymography (**Figure 6A**) and performed densitometry of each band to obtain quantitative values (**Figure 6B**). At basal level, MMP-2 and MMP-9 were not constitutively expressed in the NAL fluids of WT. However, *mev* mice showed a 10-fold increase in pro-MMP-9 and a 2-fold increase in active MMP-2 when compared with WT mice. Deficiencies of IL-4 and IL-13 resulted in a decrease in both MMPs. In contrast, both MMPs were significantly up-regulated in IFN-γ KO mice regardless of *mev* status. Expression of MMP-9 is more prominent than MMP-2 in all types of mice. From these results, MMP-2 and MMP-9 were tightly regulated by Th2/Th1 cytokines in the nasal airways.

### Increased mucus metaplasia in *mev* mice and regulation by Th2/Th1 cytokines

**Figure 7:**
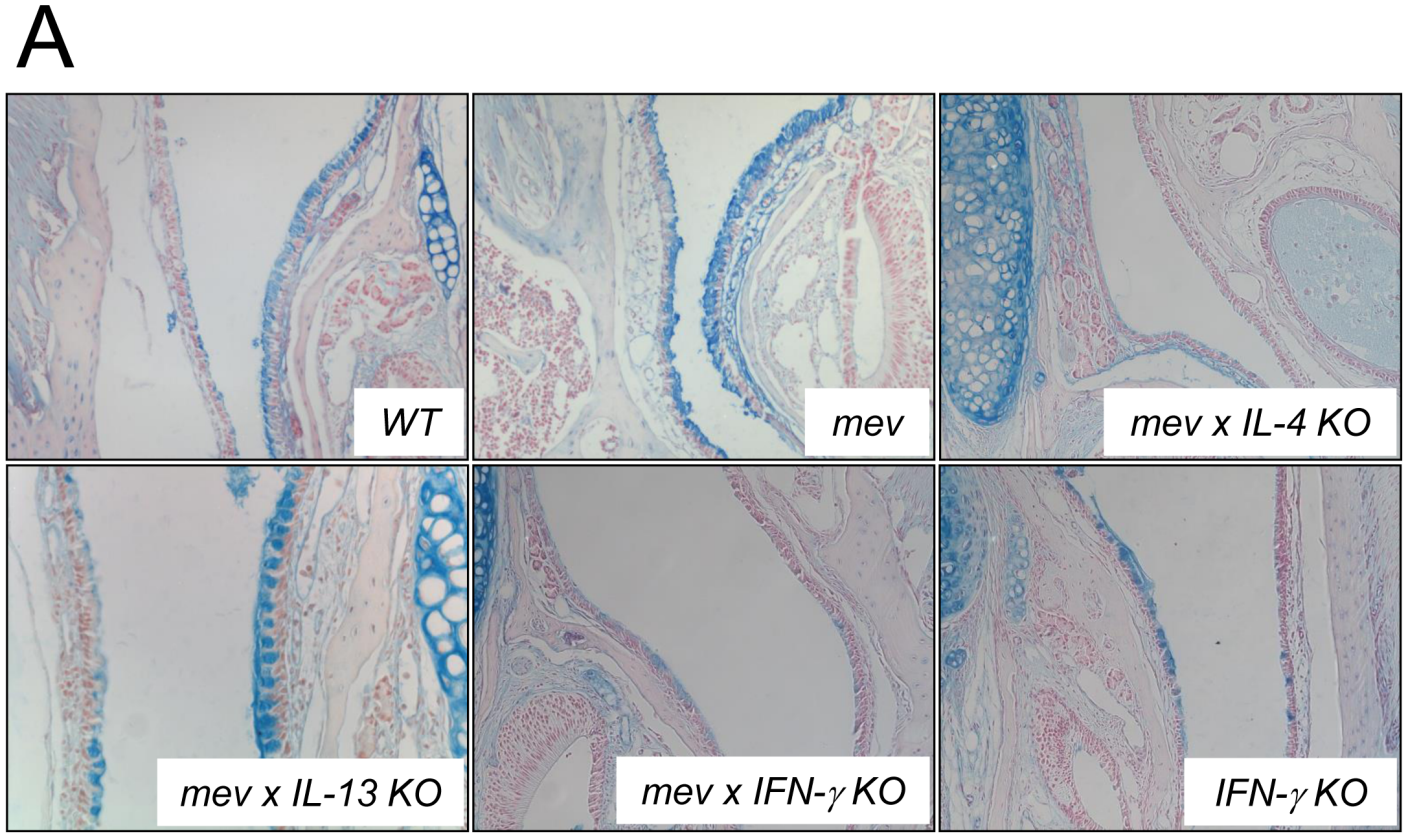
Mucus metaplasia in the nasal airways of *mev* mice. (A) Alcian blue staining of the nasal airway from *WT*, *mev*, *mev x IL-4 KO*, *mev x IL-13 KO*, *mev x IFN-γ KO*, and *IFN-γ KO* mice. Representatives of multiple samples are shown.

We next performed Alcian blue staining for goblet cell metaplasia in the nasal airways. There was baseline Alcian blue-positive staining in the airway epithelium of WT mice. The number of Alcian blue-stained goblet cells significantly increased in *mev* mice compared with WT mice (**Figure 7**). Deletion of the IL-4 and IFN-γ genes significantly decreased goblet cells in the nasal airways of *mev* mice. However, deletion of IL-13 gene had little effect on goblet cells (**Figure 7**).

## Discussion

In this study we showed evidence to support the importance of SHP-1 in the maintenance of immunologic homeostasis in the nasal airways. If the function of the SHP-1 enzyme, one of the important inhibitors of IL-4Rα signaling pathway, is decreased or lost, robust eosinophilic inflammation develops in the nasal mucosa, in the absence of any extra stimulation. This suggests that under normal conditions, SHP-1 is critical in maintaining a threshold for cell and tissue responses to normal environmental stimulation. Nasal cytology and histology also demonstrated a spontaneous eosinophilic inflammation in the *mev* mice. The mRNA expression of Th2 cytokines (IL-4 and IL-5) is up-regulated and is accompanied by a decrease in IFN-γ (**Figure 3**). In addition, eotaxin in the NAL fluids significantly increased in the *mev* mice compared with WT mice (**Figure 4**). Therefore, SHP-1 deficiency-mediated nasal inflammation is Th2-skewed. Recently, our group reported asthma-like lung inflammation and rhinitis-like nasal inflammation in the *mev* mice [17], [19]. Therefore, under normal conditions, SHP-1 is an important immune regulator in both upper and lower airways. The SHP-1 deficient *mev* mice as a Th2-dominated rhinitis model can be utilized to unravel the underlying immune mechanisms of allergic rhinitis and chronic rhinosinusitis with nasal polyposis.

Allergic airway inflammation, such as seen in asthma and allergic rhinitis, is thought to be mainly orchestrated by Th2 immunity [30]. The Th2/Th1 paradigm is a traditional concept that can explain some of the mechanisms underlying the immune homeostasis or pathologic inflammatory conditions such as asthma and autoimmune diseases [31]. Different from IL-4 and IL-5, IL-13 and IFN-γ are constitutively expressed in the nasal mucosa of unchallenged WT mice, suggesting that under normal conditions, IL-13 and IFN-γ may be present to maintain the balance of Th2/Th1 paradigm in the upper airway. This is also in contrast to the lower airways, where no IL-13 or IFN-γ can be found under normal conditions.

It is well recognized that allergic diseases are closely associated with an enhanced Th2 immune response with high levels of IL-4, IL-5, and IL-13, but accumulating evidence also implicates down-regulated Th1 immune responses in the pathogenesis of allergy [32]. Th1 lymphocytes and cytokines such as IFN-γ and IL-12 may counteract or suppress Th2 responses in allergic diseases [33]. Defective IFN-γ production predisposes toward the development of allergic diseases. For example, patients with severe asthma present significantly reduced IFN-γ production in response to allergen when compared to controls [34]. In our study, with regard to the Th1 axis, we compared the naïve immune status of the nasal airway in four different groups of mice including WT, *mev*, *mev* crossed with IFN-γ KO, and IFN-γ KO alone. The nasal airways in *mev* mice had mild eosinophilic inflammation skewed to the Th2 immune response. In line with the pulmonary findings of the *mev* mice, SHP-1 deficiency-induced nasal inflammation was down-regulated with genetic deletion of IL-4 but not significantly with deletion of IL-13. On the contrary, IFN-γ deficiency resulted in a robust eosinophilic airway inflammation with high levels of chemokines (TNF-α, CCL2, CCL5, and CXCL1; **Figure 4** **and** **5**), but interestingly, without the presence of Th2 cytokines. As shown in **Figure 2**, the *mev* mice with IFN-γ KO had even more eosinophilia and less neutrophilia compared with IFN-γ KO mice. Therefore, both SHP-1 and IFN-γ are essential in maintaining upper airway homeostasis but IFN-γ has a more prominent role.

IL-18 is another proinflammatory cytokine that promotes Th1 responses. Its role of IL-18 in the pathogenesis of allergic rhinitis is less clear than that of IFN-γ. IL-18 is up-regulated in the nasal secretions from patients with allergic rhinitis [35] or with flour allergen change [36]. It is produced by dispersed nasal polyp cells from AR patients [37], probably through promoting Th2 cytokine production. Interestingly, clinical improvement in allergic rhinitis patients with allergen specific immunotherapy (SIT) was associated with increased IL-18 in the serum or by PBMC [38]–[40]. In an animal model, Levamisole (an antihelminthic) attenuated allergic rhinitis in mice with concurrent decreases in Th2 cytokines and increases in Th1 cytokines, including IL-18 [41]. However, in a different study, improvement in symptom score and eosinophilic inflammation by steroid treatment were not correlated with changes in IL-18 in the nasal lavage fluids [42]. Thus, the role of IL-18 in AR is less clear than that of IFN-γ.

Chemokines orchestrate migration and activation of leukocyte populations under baseline and inflammatory conditions [43], [44]. The CXC chemokines mainly target neutrophils and lymphocytes, whereas the CC chemokines recruit a variety of cell types, including macrophages, eosinophils, basophils, and dendritic cells. Recently, Fulkerson, et al. showed that both CC and CXC chemokines were up-regulated in an experimental asthma model [45]. They suggested complex interactions occur between numerous chemokines in the setting of allergic airway inflammation. To know more detailed immune mechanisms in the *mev* mice, we studied the expression of both Th2 and Th1 related chemokines in the upper airways of *mev* mice.

TNF-α is a pro-inflammatory cytokine and chemokine for granulocytes including neutrophils and eosinophils [46]. Mo et al. demonstrated that TNF-α was up-regulated in OVA-induced allergic rhinitis mouse model and treatment with TNF-α inhibitor (infliximab) induced anti-allergic effects by decreasing local and systemic Th2 responses [47]. In addition to its effect on dendritic cells, neutrophils, and macrophages, GM-CSF strongly contributes to the activity of eosinophils in allergic inflammation through its capacity to prolong eosinophil survival and to generate activated eosinophils [48]. RANTES (CCL5) posses a selective chemotactic activity for eosinophils and is involved in eosinophil activation [49]. We measured the expression of pro-inflammatory cytokines and chemokines by RT-PCR and ELISA. The *mev* mice showed increased eotaxin (CCL11) concentration in the NAL fluids when compared with WT mice but not accompanied by up-regulation of MCP-1 (CCL2), RANTES (CCL5), GM-CSF, TNF-α, and KC (CXCL1). Genetic ablation of IL-4 resulted in decreased expression of eotaxin protein and GM-CSF mRNA in the *mev* mice. However, mice with IFN-γ gene deficiency showed a significantly increased expression of most of cytokines and chemokines examined. Interestingly, Th2 cytokine-deficiency showed no effect on the Th1 response with neutrophilia, but Th1 cytokine-deficiency did result in a robust Th2 response with high eosinophilia and up-regulation of chemokines, even though Th2 cytokine expression was not affected. Therefore, Th2 pathways can be considered as a ‘default pathway’ of the nasal airway. And IFN-γ may have a powerful regulatory role in chemokine expression in the nasal airway under naïve and allergic inflammation. Furthermore, lack of IFN-γ caused Th2-like inflammation occurred in the presence of increased chemokines but absence of Th2 cytokines.

MMPs have important physiological functions such as remodeling, cell migration, and immune responses [50]. In contrast to a marked peribronchial tissue eosinophilia, eosinophils in the airway were remarkably reduced in both MMP-2 and MMP-9-deficient mice [51], [52]. However, little is known about their regulatory mechanisms and signaling pathways. In our study, both MMP-2 (gelatinase A) and MMP-9 (gelatinase B) were up-regulated significantly in the *mev* mice when compared with WT mice (**Figure 6**). Deficiency of Th2 cytokines (IL-4 and IL-13) resulted in a decrease in MMPs expression when compared with the *mev* mice. In contrast to this, deficiency of Th1 cytokine (IFN-γ) led to a robust up-regulation of these MMPs. Therefore, MMPs are tightly regulated by Th2/Th1 cytokines and may play a critical role in the transepithelial clearance of inflammatory cells in the nasal airways.

In the upper and lower airways, mucus produced in the epithelium is an integral part of host defense. However, over production of mucus, as in mucus metaplasia (goblet cell hyperplasia), is one of the characteristic features of airway remodeling in allergic airway diseases that are supported by studies on animal models [53], [54]. In this study, the nasal epithelium of WT mice showed a physiological level of positively stained goblet cells. This is consistent with the presence of goblet cells in the trachea and bronchi, but in contrast to the absence of goblet cells in the small-sized airways of the lung in mice under normal conditions. However, markedly increased goblet cells were readily seen in the nasal airways of the *mev* mice (**Figure 7**) and this change is dependent on Th2 and Th1 cytokine genes. These results demonstrate that mucus production in the upper airways is closely regulated by Th2/Th1 cytokines and is very different from Th2-dominated regulation of mucus in the lower airways where IFN-γ is a major inhibitor of mucus production [33].

Our study on the *mev* mice showed that phosphatase SHP-1 plays an important role in the maintenance of immune homeostasis in the upper airways. SHP-1 may have a key role in the negative regulation of Th2 inflammation. With SHP-1 deficiency, *mev* mice display an allergic rhinitis-like phenotype, which is largely dependent on Th2 cytokines (IL-4 and IL-13) and eotaxin. There was significant up-regulation of both MMP-2 and MMP-9 in the nasal airways. In addition, our study also reveals that IFN-γ plays important regulatory roles in the Th2 nasal inflammation in the nose. A study of allergic rhinitis using a genetic approach to elucidating the basic immunological mechanisms has not been reported previously. These novel findings will help our understanding of the mechanisms of mucosal immunity and reveal some important targets to treat allergic rhinitis. Studies of a possible role of phosphatase SHP-1 in human upper airway diseases such as allergic rhinitis should be considered with SHP-1 as marker or as a potential therapeutic target.
